# The association of acute hypercarbia and plasma potassium concentration during laparoscopic surgery: a retrospective observational study

**DOI:** 10.1186/s12893-020-01034-w

**Published:** 2021-01-07

**Authors:** Laurence Weinberg, Dong-Kyu Lee, Chrisdan Gan, Damian Ianno, Alexander Ho, Luke Fletcher, Daniel Banyasz, Shervin Tosif, Daryl Jones, Rinaldo Bellomo, Dharshi Karalapillai

**Affiliations:** 1 Department of Anaesthesia, Austin Health, 145 Studley Road, Heidelberg, 3084 Australia; 2 Department of Surgery, The University of Melbourne, Austin Health, Heidelberg, 3084 Australia; 3 Department of Anesthesiology and Pain Medicine, Korea University Guro Hospital, Guro-Gu, Seoul, 08308 Republic of Korea; 4 Department of Intensive Care, Austin Health, 145 Studley Road, Heidelberg, 3084 Australia; 5 School of Public Health and Preventive Medicine, Monash University, 553 St Kilda Road, Melbourne, 3004 Australia

**Keywords:** Anaesthesia, Potassium, Hypercarbia, Laparoscopic, Surgery

## Abstract

**Background:**

It is uncertain whether increases in *P*aCO_2_ during surgery lead to an increase in plasma potassium concentration and, if so, by how much. Hyperkalaemia may result in cardiac arrhythmias, muscle weakness or paralysis. The key objectives were to determine whether increases in *P*aCO_2_ during laparoscopic surgery induce increases in plasma potassium concentrations and, if so, to determine the magnitude of such changes.

**Methods:**

A retrospective observational study of adult patients undergoing laparoscopic abdominal surgery was perfomed. The independent association between increases in *P*aCO_2_ and changes in plasma potassium concentration was assessed by performing arterial blood gases within 15 min of induction of anaesthesia and within 15 min of completion of surgery.

**Results:**

289 patients were studied (mean age of 63.2 years; 176 [60.9%] male, and mean body mass index of 29.3 kg/m^2^). At the completion of the surgery, *P*aCO_2_ had increased by 5.18 mmHg (95% CI 4.27 mmHg to 6.09 mmHg) compared to baseline values (P < 0.001) with an associated increase in potassium concentration of 0.25 mmol/L (95% CI 0.20 mmol/L to 0.31 mmol/L, P < 0.001). On multiple regression analysis, *P*aCO_2_ changes significantly predicted immediate changes in plasma potassium concentration and could account for 33.1% of the variance (r^2^ = 0.331, f(3,259) = 38.915, P < 0.001). For each 10 mmHg increment of *PaCO*_*2*_ the plasma potassium concentration increased by 0.18 mmol/L.

**Conclusion:**

In patients receiving laparoscopic abdominal surgery, there is an increase in *P*aCO_2_ at the end of surgery, which is independently associated with an increase in plasma potassium concentration. However, this effect is small and is mostly influenced by intravenous fluid therapy (Plasma-Lyte 148 solution) and the presence of diabetes.

*Trial registration* Retrospectively registered in the Australian New Zealand Clinical Trials Registry (Trial Number: ACTRN12619000716167).

## Background

Increased partial pressure of arterial carbon dioxide (*P*aCO_2_) (hypercarbia) is common in the setting of mechanical ventilation and surgery [1, 2]. The effect of hypercarbia and respiratory acidosis on plasma potassium concentrations ([K^+^]_p_) is unclear with some studies showing an increase in concentration and others showing no effect [3–10]. Further, a body of longstanding evidence indicates that the hyperkalaemia-acidaemia relationship is more complex than the relatively simplistic, but commonly accepted notion that hyperkalaemia develops due to an increase in extracellular acidity and subsequent exchange of extracellular hydrogen ions for intracellular potassium ions [8]. Challenging this theory, early studies have shown that the directional flux of potassium during acute acid–base disorders is not uniform among various tissues [11–13]. During acute respiratory acidosis, potassium was shown to move extracellularly in skeletal muscle [12, 14, 15] and liver tissue [16, 17], but intracellularly in cardiac tissue [11, 15, 18, 19]. Whilst mild hyperkalaemia intraoperatively is usually asymptomatic, high plasma levels of potassium may result in cardiac arrhythmias, muscle weakness or paralysis. Understanding this relationship has specific implications for the prevention of severe hyperkalaemia and the immediate monitoring and management of hyperkalaemia in the intra- and postoperative periods, as timely recognition and treatment of complications that arise from hyperkalaemia is imperative.

These conflicting findings, together with a lack of information from large scale studies concerning the quantitative and temporal relationships between changes in *P*aCO_2_ and [K^+^]_p_ have clinical relevance during anaesthesia. Accordingly, a retrospective study was performed to describe the association between changes in *P*aCO_2_ and acute changes in [K^+^]_p_ in patients undergoing laparoscopic surgery. Laprascopic surgery is a well-described model of carbon dioxide absorption and subsequent hypercarbia during anaesthesia [1, 2, 20–22]. The following hypotheses were made. First, in alignment with the current literature [1, 2], patients undergoing laparoscopic surgery would develop acute respiratory acidosis. Second, the development of any respiratory acidosis would cause acute respiratory acidaemia. Finally, there would be an acute rise in [K^+^]_p_ in response to the respiratory acidaemia. To test these hypotheses, patients undergoing extraperitoneal and intraperitoneal laparoscopic surgery were restrospectively studied in a university hospital.

## Methods

The Austin Health Human Research Ethics Committee approved this study (LNR/19/Austin/41) and provided a waiver for participant consent. Between February 2015 and February 2019, data was collected from the medical records of all patients undergoing extraperitoneal and intraperitoneal laparoscopic abdominal surgery at university hospital. Patients were included if they underwent surgery of greater than two hours duration, received an arterial line as part of anaesthesia care and were hospitalised for at least one postoperative night. To accurately compare changes in *P*aCO_2_ and [K^+^]_p_ during surgery to baseline values, data was collected from patients who had an arterial blood gas sampled within 15 min of induction of anaesthesia and a subsequent arterial blood gas sampled within 15 min of completion of surgery. The study was registered the study with the Australian New Zealand Clinical Trials Registry (Trial number ACTRN12619000716167; registered 13th May 2019; available at http://www.anzctr.org.au/Trial/Registration/TrialReview.aspx?id=377303&showOriginal=true&isReview=true).

Conduct of anaesthesia, was at the discretion of the treating anaesthetist and in accordance with existing protocols for patients undergoing major laparoscopic surgery at our institution. All patients received balanced crystalloid fluid therapy with the buffered fluid Plasma-Lyte 148 solution (Baxter Healthcare, Toongabbie, Australia). The physiochemical and electrolyte composition of Plasma-Lyte 148 is similar to human plasma, with a pH of 7.4 and a potassium concentration of 5 mmol/L.

During surgery, the handling and transportation of blood gas aspirates were performed using a standard hospital sampling protocol. A 3–5 mL sample of arterial blood was slowly aspirated into a 5 mL discard syringe minimising any force or strain during the aspiration process. Then, 1.0 mL of arterial blood was aspirated from the arterial line over a 2 s period and placed into a safePICO™ blood gas aspirator (Radiometer Medical Aps, Brenshel, Denmark) containing 80 IU electrolyte balanced heparin. This blood sampling technique is designed to minimise any in-vitro haemolysis, which can falsely increase values of plasma constituents, especially potassium. The safePICO aspirator has a clear 1.0 mL label designed for accurate blood sampling, which is necessary to produce reliable results.

The blood sample syringe was transported horizontally and at standard operating room temperature (22 °C) to an automated blood gas analyser (ABL800, Radiometer, Denmark). [K^+^]_p_, *P*aCO_2_, and pH were measured directly by the analyser and automatically entered into the patient’s electronic medical record. The same blood gas analyser was used for all measurements with regular calibration performed to ensure consistency in sample analysis. As a result, high analytical performance in the measurement of pH, *P*aCO_2_, [K^+^]_p_ and oximetry parameters were obtained. The system uses an automatic mixing system to obtain a homogenous sample for correct results thereby avoiding vigorous manual mixing, which can also lead to a haemolysed blood gas sample. The analyser prewarmed all samples to 37 °C prior to measurement, and the alpha-stat model was applied for all blood gases analyses.

The primary aim of the study was to describe the relationship between *P*aCO_2_ and [K^+^]_p._ To investigate the independent association between acute hypercarbia and hyperkalaemia, we collected the following preoperative patient variables: age (years), gender, body mass index (kg/m^2^), current smoker status, ASA physical status classification, history of obstructive pulmonary lung disease, presence of other comorbidities, and surgery urgency. Intraoperative variables collected included the type of laparoscopic surgery, duration of surgery and amount of intraoperative fluid administered. We also collected the ARISCAT score, adjusted for the laparoscopic surgery, which predicts the risk of pulmonary complications after surgery [23, 24].

**Fig. 1:**
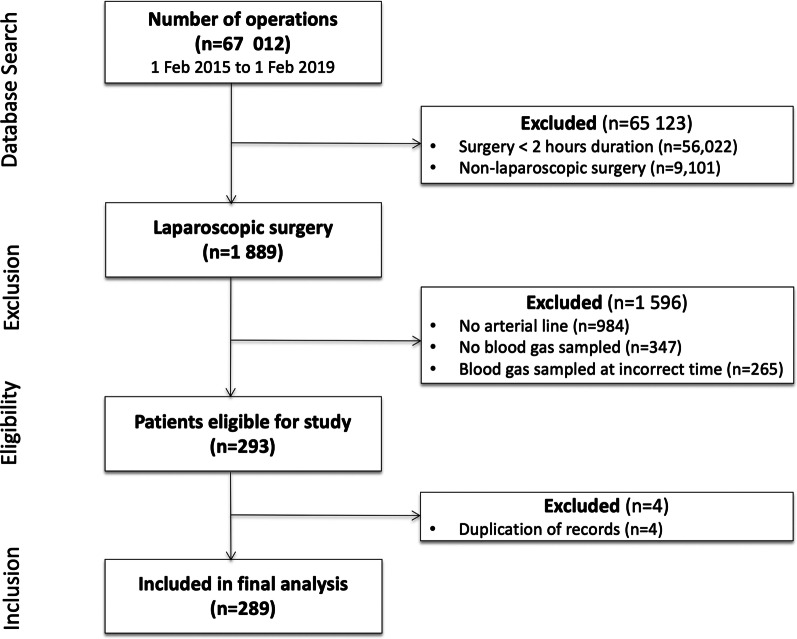
Study flow diagram

**Fig. 2:**
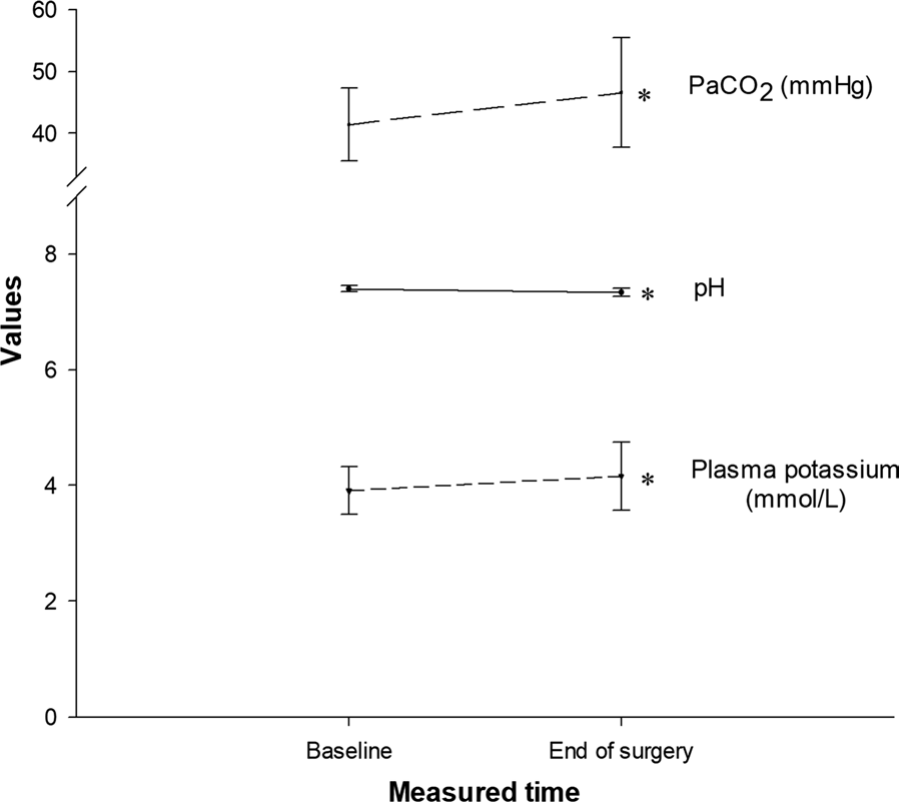
pH, partial pressure of arterial carbon dioxide (*P*aCO_2_) and plasma potassium concentrations [K^+^]_p_ changes during laparoscopic surgery. * indicates P < 0.05 with paired t-test

Inferential statistics were performed with IBM SPSS Statistics for Windows, version 23 (IBM Corp., Armonk, NY, USA). Missing value assessments were performed with R software 3.5.2 (R Development Core Team, Vienna, Austria, 2018) using “mice” and “missMDA” packages. Figure 1 was created using MS Word 365 (16.0.13127.20402) and Fig. 2 was created using SigmaPlot for Windows version 12.0 (Systat Software Inc., 2011, U.S.A).

For all continuous variables, data exploration for outliers and missing values was performed. If there were extreme outliers, winsorization was applied. Only two values from each of baseline [K^+^]_p_ and pH at the end of surgery were extreme outliers and were replaced with the most adjacent values. If the missing value rate was below 5%, we planned a complete case analysis. Normality assumptions were examined with a visual check of the quantile–quantile (Q-Q) plot. Variables of apparent violated normality assumption were treated with non-parametric statistical analysis methods.

Baseline characteristics were presented using descriptive statistics. Values at baseline and at the end of surgery were compared using the paired t-test. To investigate the relationship between the magnitude of *P*aCO_2_ changes, [K^+^]_p_ changes, and patient characteristics, correlation analysis was performed. For the relationship between the magnitude of *P*aCO_2_ changes and [K^+^]_p_ changes, visual checking of the scatter plot and curve-fit analysis using regression were performed with various types of equations. Based on these results, linear regression was performed to clarify the relationship between the magnitude of *P*aCO_2_ changes and [K^+^]_p_ changes, and other patient characteristics.

To establish the final model, the stepwise selection method was used, and assumptions of linear regression were tested with a histogram and a Q-Q plot for multivariate normality, correlation matrix and variance inflation factors for multicollinearity, Durbin-Watson statistics for autocorrelation, and visual checking of the scatter plot for each independent variable for homoscedasticity. Regression diagnostics were performed with the normality test of residuals. Finally, the characteristics of patients who presented with hypercarbia were also evaluated to enhance the understanding of risk factors for intraoperative CO_2_ retention. All data were presented with mean ± SD, number (percentile), or median (IQR). The statistically inferred values were expressed as mean (95% confidence intervals [CI]) with corresponding effect sizes, and *P* values. A two-tailed *P* value of below 0.050 was considered statistically significant.

## Results

A graphical representation of patients undergoing surgery during the study period is presented in Fig. 1. Overall, 1889 patients underwent laparoscopic surgery. Of these, 1596 patients were excluded (no arterial line: n = 984, no blood gas analysis performed: n = 347, blood gas sampled at incorrect times: n = 265). Two hundred and ninety-three patients fulfilled the study inclusion criteria. Four patients were excluded due to duplication of data from the records. A total of 289 patients were included in the final statistical analysis (Additional file 1: De-identified database).

The mean (SD) age of patients was 63.2 ± 11.5 years; 176 (60.9%) patients were male, and the mean (SD) body mass index was 29.3 ± 6.8 kg/m^2^. The mean (SD) corrected ARISCAT score was 29.5 ± 11.8, and 51 (17.6%) patients were current smokers. Patient comorbidities and baseline characteristics are presented in Table 1. Most surgeries were elective, and the median (IQR) duration of surgery was 184 min (137.3 min to 240.0 min). For all 6 patients who underwent emergency surgery, the admission diagnosis was acute lower gastrointestinal bleeding. No patient had acute peritonitis and all patients underwent definitive surgery after inpatient optimisation.

**Table 1:** Preoperative and intraoperative variables

Parameters	Patients (n = 289)	Missing rate (%)
Age (years)	63.2 ± 11.5	0.00
Male gender	176 (60.9%)	0.00
Body mass index (kg/m^2^)	29.3 ± 6.8	0.35
Corrected ARISCAT score	29.5 ± 11.8	4.84
Current smoker	51 (17.6%)	0.00
American Society of Anesthesiologists status		
Class 1	21 (7.3%)	
Class 2	124 (42.9%)	2.08
Class 3	129 (44.6%)	
Class 4	9 (3.1%)	
No of patients with ≥ 1 chronic disease	214 (74.0%)	0.00
Types of chronic disease^*^		
Obstructive lung disease	84 (29.1%)	
Type II diabetes mellitus	57 (19.7%)	
Essential hypertension	149 (51.6%)	
Renal disease (creatinine > 120 *u*mol/L)	25 (8.7%)	0.00
Hepatic disease (bilirubin > 20 *u*mol/L)	25 (8.7%)	
Coronary artery disease	46 (15.9%)	
Emergency surgery	6 (2.1%)	0.00
Types of laparoscopic procedures		
Anterior resection	61 (21.1%)	
Left or right hemicolectomy	60 (20.8%)	
Small bowel resection	2 (0.7%)	
Intestinal bypass	2 (0.7%)	0.00
Adrenalectomy	4 (1.4%)	
Colostomy or adhesiolysis	6 (2.1%)	
Cholecystectomy	10 (3.5%)	
Diaphragmatic hernia repair	1 (0.3%)	
Fundoplication	5 (1.7%)	
Sleeve gastrectomy gastric bypass	17 (5.9%)	
Splenectomy	2 (0.7%)	
Pancreatectomy	1 (0.3%)	
Hernia repair	7 (2.4%)	
Liver resection	20 (6.9%)	
Nephrectomy	45 (15.6%)	
Radical prostatectomy	35 (12.1%)	
Pyeloplasty	1 (0.3%)	
Rectopexy	1 (0.3%)	
Removal of pelvic or peritoneal mass	3 (1.0%)	
Exploratory procedures	6 (2.1%)	
Duration of surgical procedure (min)	184.0 (137.3 to 240.0)	0.35
Intraoperative fluid administration (l)	2.0 (1.0 to 2.1)	1.73

Values are expressed as mean ± standard deviation or median (interquartile range) for continuous variables, number (percentile) for categorical variables. Missing data rate of each variable is presented.

* Some patients had > 1 chronic disease

Intraoperatively, no patient received intraoperative potassium supplementation except for that contained in Plasma-Lyte 148. The median (IQR) volume of Plasma-Lyte 148 solution administered was 2.0 L (1.0 L to 2.1 L), with an approximate estimated infusion rate of 11 mL/min. Nine patients (3.8%) received an intraoperative blood transfusion. The median (interquartile range) number of packed red blood cell units transfused was 1 unit (1 unit to 3 units). No patient received a massive blood transfusion. The maximum number of red blood units transfused was three.

Comparisons of pH, *P*aCO_2_ and [K^+^]_p_ at baseline and at the end of surgery are presented in Table 2. At the completion of the surgery, *P*aCO_2_ had increased by 5.18 mmHg (95%CI, 4.27 mmHg to 6.09 mmHg) compared to baseline values (P < 0.001, Cohen’s *d* = 1.26,). pH also decreased reciprocally to the increased *P*aCO_2_ (P < 0.001, Cohen’s *d* = 0.99), with a similarly large effect size observed. [K^+^]_p_ increased by 0.25 (95% CI: 0.20 to 0.31) mmol/L at the end of surgery compared to baseline values (*P* < 0.001, Cohen’s *d* = 0.03). The effect size of this change was small. Before and after changes in these variables are presented graphically in Fig. 2. Bicarbonate and standard base excess values remained within normal laboratory limits at all time points (Table 2).

**Table 2:** Comparisons of pH, partial pressure of arterial carbon dioxide (*P*aCO_2_) and plasma potassium concentrations [K^+^]_p_ between baseline and end of surgery

Variable	Baseline	End of surgery	Paired differences	*P* value	Cohen’s *d*
pH	7.401 ± 0.053	7.34 ± 0.069	− 0.061 (− 0.068 to − 0.054)	< 0.001*	0.99
*P*aCO_2_ (mmHg)	41.44 ± 5.84	46.61 ± 8.83	5.18 (4.27 to 6.09)	< 0.001*	1.26
[K^+^]_p_ (mmol/L)	3.911 ± 0.416	4.162 ± 0.591	0.251 (0.197 to 0.305)	< 0.001*	0.03
Bicarbonate (mmol/L)	24.96 ± 3.58	23.90 ± 3.61	1.06 (0.54 to 1.58)	< 0.001*	0.19
Standard base excess (mmol/L)	0.87 ± 2.97	− 0.93 ± 2.74	1.80 (1.44 to 2.16)	< 0.001*	0.24

Paired differences were presented with 95% confidence intervals. The effect size is presented with Cohen’s *d*.

* Indicates P < 0.05 with paired t-test

Correlation analysis between *P*aCO_2_ changes, [K^+^]_p_ changes and other baseline patient characteristics are presented in Table 3. Increases in *P*aCO_2_ correlated weakly with increasing BMI (r = 0.12, *P* = 0.049), and higher [K^+^]_p_ were associated with type II diabetes mellitus (rho = 0.13, *P* = 0.023). According to curve-fit analysis results, a linear model (R^2^ = 0.217, *P* < 0.001) was suitable because quadratic and squared models resulted in the same proportion of the variance for the [K^+^]_p_ change associated with *P*aCO_2_ changes (R^2^ = 0.217, *P* < 0.001 for the quadratic and squared models).

**Table 3:** Correlation analysis between partial pressure of arterial carbon dioxide (*P*aCO_2_) changes, plasma potassium concentrations [K^+^]_p_ changes and other parameters

Variables	*P*aCO_2_ changes		[K^+^]_p_ changes	
	Coefficient	P value	Coefficient	P value
Age	− 0.03	0.642	− 0.01	0.807
ASA classification	− 0.06	0.315	0.04	0.546
BMI	0.12*	0.049	0.03	0.632
Current smoking status	0.06	0.347	0.03	0.571
Lung disease	0.06	0.348	0.09	0.132
Coronary disease	− 0.04	0.553	− 0.01	0.829
Type II diabetes mellitus	− 0.04	0.534	0.13*	0.023
Essential hypertension	− 0.05	0.394	0.02	0.679
Renal disease	0.03	0.576	− 0.07	0.215
Hepatic disease	− 0.03	0.568	0.09	0.128
Elective / Emergency operation	0.08	0.161	− 0.02	0.735
Duration of surgery	0.02	0.783	0.10	0.102
Corrected ARISCAT score	0.06	0.356	0.06	0.337
Intraoperative fluid amount	0.01	0.928	0.10	0.113

Coefficients are presented as Pearson correlation for continuous variables or Spearman’s rho for categorical variables

* Indicates P < 0.05

Multiple regression analysis evaluating whether *P*aCO_2_ changes during surgery significantly predicted immediate changes in [K^+^]_p_ at completion of surgery demonstrated that a final fitted regression model could predict 33.1% of the observed variance in *P*aCO_2_ (R^2^ = 0.331, F(3,259) = 38.915, *P* < 0.001). Regression analysis showed that *P*aCO_2_ changes, intraoperative Plasma-Lyte 148 administration volume, and a history of type II diabetes mellitus significantly predicted changes in [K^+^]_p_ at completion of surgery (β = 0.018 (95% CI: 0.011 to 0.025), *P* < 0.001; β = 0.061 (95% CI: 0.031 to 0.091), P < 0.001; β = 0.154 (95% CI: 0.023 to 0.284), *P* = 0.021 respectively). There was no association between [K^+^]_p_ and duration of surgery (*P* = 0.102, Table 4).

**Table 4:** Coefficients estimated using linear regression for predicting plasma potassium concentrations [K^+^]_p_ changes

Variables	Coefficient	P value
Included in the final regression model		
* P*aCO_2_ changes	0.018 (0.011‒0.025)	< 0.001
Intraoperative Plasma-Lyte 148 administration volume	0.061 (0.031‒0.091)	< 0.001
Type II diabetes mellitus	0.154 (0.023‒0.284)	0.021
Excluded in the final regression model		
Age	0.084	0.464
American Society of Anesthesiologists status	0.029	0.683
Body mass index	0.046	0.703
Smoking history	− 0.008	0.889
Obstructive lung disease	0.058	0.348
Coronary artery disease	− 0.041	0.464
Hypertension	0.014	0.847
Renal disease (creatinine > 120 *u*mol/L)	− 0.102	0.058
Hepatic disease (bilirubin > 50 *u*mol/L)	0.07	0.199
Emergency surgery	− 0.036	0.497
Duration of surgical procedure	0.156	0.235
Corrected ARISCAT score	0.082	0.475

Coefficient values are expressed with 95% confidential intervals when corresponding variable is included in the final regression model

Thus, for each 10 mmHg increment of *P*aCO_2_ from baseline during surgery, [K^+^]_p_ increased by 0.18 mmol/L. Furthermore, [K^+^]_p_ increased by 0.061 mmol/Lfor each litre of Plasma-Lyte 148 administered, and patients with type II diabetes mellitus experienced a 0.154 mmol/L greater rise in [K^+^]_p_ compared to those without.

## Discussion

In a single centre retrospective study describing the association of acute changes in *P*aCO_2_ with acute changes in [K^+^]_p_ in patients undergoing laparoscopic surgery, CO_2_ pneumoperitoneum induced small but significant increases in *P*aCO_2_ and small but significant decreases in pH during surgery. Moreover, [K^+^]_p_ increased slightly but significantly at the end of surgery compared to baseline. After adjustment for multiple potential confounders, for every 10 mmHg increment in *P*aCO_2_ from baseline, [K^+^]_p_ increased by almost 0.2 mmol/L. Finally, changes in [K^+^]_p_ were additionally positively affected by the amount of Plasma-Lyte 148 administered and the presence of type II diabetes mellitus. Given the magnitude of the changes observed, our findings imply that any acute hypercarbic or hyperkalaemic state induced by laparoscopic surgery is probably of limited clinical significance.

Our findings imply that during surgery clinicians should not ascribe large changes in [K^+^]_p_ to hypercarbia. Other aetiological factors should be considered as a more likely cause of hyperkalaemia. Such differentials should include renal failure, non-renal and endocrine causes, medications and surgical factors. The main hormonal system regulating renal potassium excretion is the renin–angiotensin–aldosterone system. Medications used in the perioperative setting that inhibit this system include angiotensin converting enzyme inhibitors, angiotensin II receptor blockers, nonsteroidal anti-inflammatory drugs, and adrenergic beta-antagonists. Other common anaesthesia medications known to cause hyperkalaemia during surgery include suxamethonium, beta-blockers, digoxin, mannitol and some intravenous penicillins (high potassium content). Finally, surgical causes that can result in the release of potassium from injured cells include ischaemia–reperfusion injury, rhabdomyolysis from surgical associated muscle damage, and high-volume blood transfusion. In many patients, during surgery the cause of hyperkalaemia is multifactorial.

Our findings concur with the landmark study by Finsterer et al. [8], who prospectively investigated the effects of hypercarbia on [K^+^]_p_ in 17 patients undergoing major abdominal surgery. The authors induced a model of hypercarbia (mean *P*aCO_2_ of 71 mmHg) by removing the soda-lime absorbers from the anaesthesia circuit, and without changing minute volume ventilation and adding CO_2_ to the inspiratory gas to obtain elevated end-tidal CO_2_ concentrations. *P*aCO_2_ rose by about 30 mmHg resulting in a decrease in pH of 0.2–0.25. A linear relationship between *P*aCO_2_ and [K^+^]_p_ was observed and the authors reported that [K^+^]_p_ increased by 0.30 mmol/L after 90 min of induced hypercarbia. Our findings are also aligned with others [25] showing that *P*aCO_2_ and [K^+^]_p_ are related, even with minor degrees of hypercarbia. Hassan et al. [25], reported that [K^+^]_p_ increased by 0.39 mmol/L per every 7.5 mmHg increase in *P*aCO_2_, a greater increase compared to the present study. Hassan’s findings were almost identical to that reported by Edwards et al. [26], who showed that [K^+^]_p_ increased by 0.4 mmol/Lper 10 mmHg change in *P*aCO_2_, a doubling of what was observed in the present study.

Our findings however are at variance with the case series of 24 patients undergoing cardiac surgery who were exposed to a 15 min period of apnoeic or low tidal volume ventilation [9]. Over this time period, *P*aCO_2_ increased from 43.6 mmHg at baseline to 83.9 mmHg with [K^+^]_p_ increasing marginally from 4.16 mmol/L at baseline to 4.28 mmol/L at 15 min. This study was limited by a small sample size and exposure to significant hypercarbia for only a 15 min duration. Similarly, Natalani et al. [10] compared the acute changes in [K^+^]_p_ in acutely hypercapnic patients undergoing rigid bronchoscopy. The sampling time to evaluate the effects of *P*aCO_2_ on hyperkalaemia was also of short duration (20 min), and acute respiratory acidosis did not affect [K^+^]_p_. These findings suggest that there appears to be both a quantitative and temporal relationship between changes in [K^+^]_p_ and hypercarbia under anaesthesia, and that acute hypercarbia of short duration may have less of an effect on [K^+^]_p_ compared to prolonged exposure. As there was no significant association between [K^+^]_p_ and duration of surgery in this study, and only patients who had a surgical duration of two hours were included, it is likely that the temporal onset of [K^+^]_p_ change due to a change in *P*aCO_2_ is less than two hours.

Our study has several strengths. Data on a relatively large sample size was provided and a detailed description of the acute effects of hypercarbia and its associations with [K^+^]_p_ during laparoscopic surgery were analysed. *P*aCO_2_, [K^+^]_p_, bicarbonate and standard base excess values were collected directly from arterial blood samples and were not amenable to ascertainment bias or derivation. The timing of blood gases in relation to the start and completion of surgery was manually cross-checked with the patients’ medical records by two independent investigators, allowing for accurate confirmation of these data points at the start and at completion of surgery. The large dataset examines the impact of laparoscopic surgery on *P*aCO_2_ and [K^+^]_p_ by directly measuring *P*aCO_2_ and [K^+^]_p_ at baseline and at the completion of surgery, allowing a greater understanding of the temporal relationship of changes in [K^+^]_p_ and *P*aCO_2_ to be observed.

Our study has several limitations. It was a single centre study, limiting the external validity of our findings. However, given laparoscopic surgery with CO_2_ insufflation is utilised worldwide, our findings are likely relevant and externally generalisable. We cannot extrapolate our results to shorter surgical times, to paediatric patients or to patients at high risk of developing hyperkalaemia (renal failure patients, rhabdomyolysis, tumour lysis syndrome etc.). However, the biological principles and physiochemical changes described are likely to apply to all hypercarbic patients undergoing general anaesthesia. As only two blood gases were sampled, the exact onset and rate of change of the findings observed cannot be evaluated. This study was not designed to determine whether there was an association between changes in *P*aCO_2_ or [K^+^]_p_ and the occurrence of detrimental clinical outcomes. Similarly, we did not evaluate the association between changes in *P*aCO_2_ or [K^+^]_p_ with specific medications that are known to be associated with hyperkalaemia such as preoperative use of certain antihypertensive medications e.g. angiotensin-converting enzyme inhibitors, angiotensin II receptor blockers, and/or potassium-sparing diuretics. This data was unable to be collected and we acknowledge this as a limitation of the study. However, this study was designed to evaluate the change in *P*aCO_2_ from baseline rather than the absolute value of [K^+^]_p_ at baseline, which is more likely to be effected by these medications. As the anaesthesia records in our institution are hand-written, and not part of the hospital’s electronic medical record system, corresponding end-tidal CO_2_ values were not able to be collected retrospectively. However, the focus of this study was primarily on the association of *P*aCO_2_ and [K^+^]_p_, rather than end-tidal CO_2_ and acid–base responses to an increased *P*aCO_2_. For all cases the initial CO_2_ insufflation occurred at a rate of 1 to 5 L/min to achieve an intraabdominal pressure of 12 to 15 mmHg. We used a constant CO_2_ flow of approximately 200 to 400 mL/min to maintain pneumoperitoneum perioperatively. Given this standard practice for all patients, we cannot make any inferences about the relationship of intraabdominal pressure and changes in *P*aCO_2_. Finally, due to the heterogeneity of clinical precipitants for potassium disorders, and the observational nature of this study, our findings are merely associations and do not infer causation.

## Conclusion

During laparscopic surgery, for every 10 mmHg increase in *P*aCO_2_ from baseline, [K^+^]_p_ increased by almost 0.2 mmol/L. [K^+^]_p_ was further elevated with the administration of the crystalloid solution Plasma-Lyte 148 and the presence of type II diabetes mellitus. Clinicians should not ascribe larger changes in [K^+^]_p_ to hypercarbia in this setting and should consider other aetiological factors in their differential diagnosis.

## Supplementary information

**Additional file 1: Table S1.** Database_Final_CO2_Study_OctR4_2020.

## Data Availability

The deidentified datasets analysed during the current study are available from the corresponding author on reasonable request.

## Abbreviations

*P*aCO_2_: Partial pressure of arterial carbon dioxide
CO_2_: Carbon dioxide
CI: Confidence interval
[K^+^]_p_: Plasma potassium concentrations
Q-Q: Quantile–quantile
